# Increased Cerebrospinal Fluid S100B and NSE Reflect Neuronal and Glial Damage in Parkinson’s Disease

**DOI:** 10.3389/fnagi.2020.00156

**Published:** 2020-07-22

**Authors:** Ewa Papuć, Konrad Rejdak

**Affiliations:** Department of Neurology, Medical University of Lublin, Lublin, Poland

**Keywords:** Parkinson’s disease, cerebrospinal fluid, neuronal damage, glial damage, biomarkers, S100B protein, neuron specific enolase

## Abstract

**Introduction**: The diagnosis of Parkinson’s disease (PD) mainly relies on clinical manifestation, but may be difficult to make in very early stages of the disease, especially in pre-motor PD. Thus, there is great interest in finding a biomarker for PD. Among diagnostic biomarkers, the most promising molecules are those which reflect the pathophysiological mechanisms of the disease. Until now, only α-synuclein, a classical CSF Alzheimer’s disease biomarker, and neurofilament light (NFL) chains have turned out to be helpful in differential diagnosis between PD and healthy control subjects.

**Aim**: To assess whether CSF molecules related to some pathological processes present in PD might be of interest in the diagnosis of PD and whether they correlate with disease severity.

**Methods**: CSF levels of S100B and neuron-specific enolase (NSE) were measured in 58 PD patients and in 28 healthy control subjects. Correlations were determined between the levels of these CSF molecules and measures of disease severity (Hoehn–Yahr scale and UPDRS part III), as well as disease duration and levodopa dose.

**Results**: CSF S100B and CSF NSE were both significantly increased in PD subjects vs. healthy controls (*p* = 0.007 and *p* = 0.00035, respectively). CSF S100B was significantly positively correlated with measures of disease severity (H-Y score and UPDRS part III), as well as disease duration (*p* < 0.05). No correlation was found between CSF NSE levels and disease severity or disease duration (*p* > 0.05). CSF S100B levels alone provided a relatively high discrimination (AUC 0.77) between PD and healthy controls, with 60.7% sensitivity and 88.5% specificity (*p* < 0.001) at a cut-off value of 123.22 pg/ml. Similarly, CSF NSE levels alone provided a relatively high discrimination (AUC 0.775) between PD and healthy controls, with 78.6% sensitivity and 74.1% specificity at a cut-off value of 51.56 ng/ml (*p* < 0.001).

**Conclusions**: Our results show that both CSF S100B and CSF NSE seem to be promising markers of the axonal and glial degeneration present in PD. Additionally CSF S100B may be a promising marker of PD progression.

## Introduction

Parkinsonian syndromes involve a large group of different disorders, with most frequent Parkinson’s disease (PD), but also rarer atypical parkinsonisms. Differential diagnosis of these disorders may be sometimes challenging, especially in very early stages of the disease. Presently, the diagnosis of PD is based on the clinical manifestation and the exclusion of atypical features suggestive of atypical parkinsonism.

That is why the search for blood and CSF biomarkers which could be helpful in PD diagnosis is so crucial. Other currently available biomarkers for PD are either expensive or not easily available. Additionally, there is no specific CSF biomarker for PD, to date. This is partially due to the mixed pathology of PD, with different underlying pathological processes (Sabbagh et al., 2009; Irwin et al., 2012). The most crucial pathological processes present in PD include α-synuclein depositions in midbrain with later spread to the limbic system, and neocortex, as well as neurotransmitter abnormalities, including mainly acetylcholine and dopamine deficits. PD subjects with dementia may have additional neuropathological findings consistent with Alzheimer’s disease pathology (Galvin et al., 2006). Interestingly, about 40% of Parkinson’s disease dementia (PDD) cases also fulfill the diagnostic criteria for Alzheimer’s disease (Mattila et al., 2000). All the above numerous pathological abnormalities make it difficult to find one biochemical biomarker characteristic for PD.

Different biomarkers were examined in order to enable distinction between PD and atypical parkinsonism, especially in early stages of the disease (Katayama et al., 2020). Despite numerous studies, which have focused on identifying multiple brain derived CSF markers for PD, to date none of them have turned out to be efficacious. Additionally, presently there is no possibility to assess degree of neuronal loss after PD diagnosis has been made. That is why a biochemical biomarker that can help with the assessment of PD progression would be of great value.

In this study, we measured markers of neuronal and glial degeneration in PD, to check whether these two pathological processes present in PD, are reflected by changes of these molecules in CSF. The detailed aim of our study was also to assess CSF levels of S100B as a marker of glial activation in PD, and CSF levels of neuron-specific enolase (NSE) as a marker of neuronal damage.

### S100B

S100B is a calcium binding protein mostly produced and released by astrocytes in the central nervous system (CNS; Donato, 2001). This protein is a marker of glial activation and degeneration. CSF S100B elevations may occur in different brain disorders, before major changes in the brain occur (Sorci et al., 2010).

Increased CSF or serum S100B levels were found in different acute and chronic brain disorders, such as traumatic brain injury (Herrmann et al., 1999; Ingebrigtsen et al., 1999), ischemic stroke (Monbailliu et al., 2017) or Alzheimer’s disease (Christl et al., 2019).

This protein has been reported to be a very sensitive marker of glial damage and rise prior to any detectable changes in intracerebral pressure, changes in neuroimaging, and prior to neurological signs and symptoms.

Data from animal models of PD revealed increased expression of S100B in glial cells of mice exposed to MPTP, which gave rise to the hypothesis that this protein may be involved in the pathogenesis of PD (Muramatsu et al., 2003). There is also evidence that the initiation and progression of PD is mainly driven by the glial cells (Halliday and Stevens, 2011).

There is also increasing evidence from human studies that S100B acts as a protein of neuronal damage not only in inflammatory, but also in neurodegenerative disorders with concomitant underlying neuroinflammatory process (Sathe et al., 2012). Sathe et al. (2012) found increased expression of S100B in post-mortem examination of substantia nigra of six PD subjects. Additionally, Sathe et al. (2012) revealed that genetic ablation of S100B reduces microglial response, which strengthens the hypothesis that S100B activation is involved in the neuroinflammation present in PD.

Since S100B has not been widely investigated in PD so far, and in the light of the above data we decided to measure CSF levels of S100B as a marker of glial activation.

### NSE

The NSE is a cytoplasmic enzyme, whose isoform can be found in neurons, and in neuroendocrine cells. Since NSE is not physiologically secreted, its increased levels in human serum and CSF levels are considered a marker of neuronal damage (Isgrò et al., 2015). Its elevated levels were reported after traumatic brain injury (Herrmann et al., 1999), in different types of stroke (Isgrò et al., 2015), and in Alzheimer’s disease (Christl et al., 2019). Recent CSF S100B assessment in AD subjects revealed its strong correlation with CSF NSE, but not with other CSF biomarkers typical of AD like total tau protein, hyperphosphorylated tau protein (p-tau) and amyloid β1–42 (Aβ1–42; Christl et al., 2019).

As NSE may be treated as non-specific marker of neuronal damage in different neurodegenerative disorders, we decided to assess its CSF levels in PD.

## Materials and Methods

CSF samples were obtained by lumbar puncture, processed and stored according to the consensus protocol for the CSF biobanking (Teunissen et al., 2009).

Study inclusion period was between Nov 2018 and Dec 2019, and all PD patients, who gave informed consent to participate in the study, were assessed in Unified Parkinson’s Disease Rating Scale (UPDRS; Fahn and Elton, 1987) and in Hoehn-Yahr score (H-Y; Jankovic et al., 1990) by the neurologist specialized in movement disorders. Patients’ cognitive status was assessed in Mini Mental State Examination (MMSE). PD patients with dementia were not included into the study. Out of 85 non demented PD subjects consecutively admitted to our department, 58 PD subjects gave informed consent to participate in this study and to undergo lumbar puncture. The diagnosis of PD was confirmed by two neurologists experienced in movement disorders (EP or KR), according to current diagnostic criteria (Postuma et al., 2015). Exclusion criteria were Parkinson’s disease dementia.

CSF was obtained by lumbar puncture and collected in polypropylene tubes. CSF was routinely assessed for cell counts, protein and glucose level, centrifuged, and stored at −80°C within 2 h from the lumbar puncture, according to the current guidelines (Teunissen et al., 2009).

S100B in CSF levels were measured by a commercially available ELISA system (Merck Millipore, Burlington, MA, USA). S100B levels were presented in picograms per milliliter (pg/ml).

CSF NSE levels were measured using human Elisa kit (Demeditec). NSE levels were expressed in nanograms per milliliter (ng/ml). All samples were analyzed in duplicates.

Additionally, we collected CSF samples from 28 healthy controls matched for age and gender. Control subjects were also patients admitted to the Department of Neurology, who required lumbar puncture for different reasons (e.g., severe headache to exclude subarachnoid hemorrhage or meningitis) and had pathological processes in the CNS excluded.

The study was approved by the local ethics committee of Medical University of Lublin, Poland (consent ID KE-0254/292/2017), and all study participants gave written informed consent for participation in the study.

### Statistical Analysis

Comparison between groups was made by using a parametric *t*-test for NSE, and a non-parametric Mann Whitney test for S100B. Correlations are presented with Pearson’s coefficient, for NSE with age, disease duration, disease severity (assessed in H-Y scale and in UPDRS part III) and levodopa dose as well as levodopa equivalent; and with nonparametric correlation coefficient rho Spearman’s for all S100B correlations. *P* < 0.05 was considered statistically significant.

To establish the optimal cut-off point the Youden index was used, which maximizes the sum of sensitivity and specificity.

Descriptive statistics were calculated for every group. Significances (*p*) were tested by the GraphPad Prism, version 7.0 (GraphPad Software Inc.).

## Results

Measurements of various CSF marker levels were performed on 58 patients with PD without cognitive decline, and 28 cognitively healthy patients. Clinical characteristics of the investigated subjects are displayed in Table 1.

**Table 1:** Clinical and biochemical characteristics of the study group.

	Parkinson’s disease patients (*n* = 58)	Healthy control group (*n* = 28)	Difference (*p*)
Age (years), mean ± SD	65.18 ± 8.54	58.14 ± 13.71	*p* > 0.05
Gender (M/F)	30/28	13/15	*p* > 0.05
MMSE (mean ± SD)	26.46 ± 2.46	28.86 ± 1.11	*p* > 0.05
Pure L-dopa daily dose (mg)	732.14 ± 325, 81	NA	NA
L-dopa daily equivalent (mg)	1,127.14 ± 405.75	NA	NA
Hoehn-Yahr scale	3.2 ± 0.89	NA	NA
UPDRS III “ON”	47.57 ± 10.60	NA	NA
UPDRS III “OFF”	63.57 ± 10.00	NA	NA
Disease duration (years)	5.61 ± 2.15	NA	NA
CSF NSE level (ng/ml) Mean ± SD (Range)	55.40 ± 7.17 (38.94–67.89)	45.88 ± 10.91 (25.78–70.88)	*p* = 0.00035
CSF NSE level (ng/ml), median (Interquartile range)	54.74 (51.75–61.01)	45.8 (38.09–51.55)	-
CSF S100B level (pg/ml), mean ± SD (Range)	133.52 ± 54.72 (37.52–54.72)	114.86 ± 120.69 (552.94–6,340.69)	-
CSF S100B level (pg/ml), median (Interquartile range)	125.05 (88.93–172.26)	84.74 (50.75–110.23)	*p* = 0.0067

*MMSE, Mini Mental State Examination; NA, not applicable. Demographic data are presented as means with standard deviation (SD). CSF markers levels are presented both as mean ± SD and as median with interquartile range. NA, not applicable*.

The PD and healthy controls group were matched for gender and age, no statistically significant differences between investigated groups were found in terms of age or gender (*p* > 0.05).

### S100B

**Figure 1:**
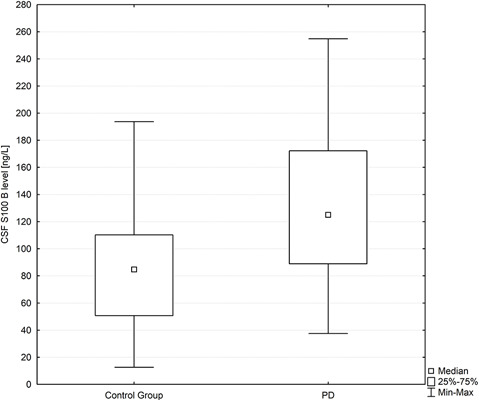
Box plot of CSF S100B levels in PD subjects and healthy controls. The central square point in each box indicates the median, box edges mark the first and third quartiles, and limits of the vertical lines show ranges. PD, Parkinson’s disease.

CSF S100B levels were significantly higher in PD patients in comparison to healthy controls. Since a non parametric test was applied for group comparisons, data are presented as median with interquartile range [125.05 (88.93–172.26) vs. 84.74 (50.75–110.23) ng/L; *p* = 0.007; Figure 1].

Among PD patients a significant positive correlation was found between CSF S100B levels and disease severity assessed in H-Y scale (*R* = 0.5917, *p* = 0.01).

A positive correlation was also found between CSF S100B levels and disease duration (*R* = 0.3838; *p* = 0.044).

No correlation was found between CSF S100B levels and age (*p* = 0.07). No correlation was also found between CSF S100B levels and levodopa dose (*p* = 0.34) or levodopa equivalent (*p* = 0.34; Table 2).

**Table 2:** Rho Spearman correlation coefficients between CSF S100B levels and clinical variables (stage of the PD on Hoehn-Yahr scale, UPDRS “ON” state, UPDRS “OFF” state, and disease duration) in a group of PD patients. For correlations of CSF NSE with clinical variables r Pearson correlation coefficients were presented.

Clinical variable	CSF S100B levels (ng/ml)	*p*	CSF NSE (pg/ml)	*p*
Disaese severity (Hoehn-Yahr scale)	0.46	*P* = 0.008	−0.01	*p* > 0.05
PD duration	0.37	*p* > 0.05	0.16	*p* > 0.05
UPDRS “ON”	0.19	*p* > 0.05	−0.05	*p* > 0.05
UPDRS “OFF”	0.36	*p* > 0.05	−0.07	*p* > 0.05

CSF S100B levels alone provided a high discrimination (AUC 0.77) between PD and healthy controls, with 60.7% sensitivity and 88.5% specificity (*p* < 0.001; Figure 3). A cut-off value to differentiate between PD and healthy subjects was 123.22 pg/ml (Youden index).

**Figure 2:**
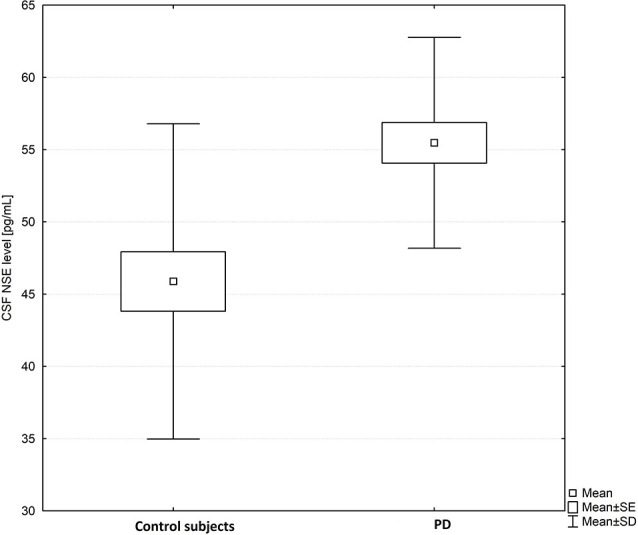
Box plot of CSF neuron-specific enolase (NSE) levels in PD subjects and healthy controls. The central square point in each box indicates the mean, box edges mark standard deviation, and limits of the vertical lines show ranges. PD, Parkinson’s disease.

**Figure 3:**
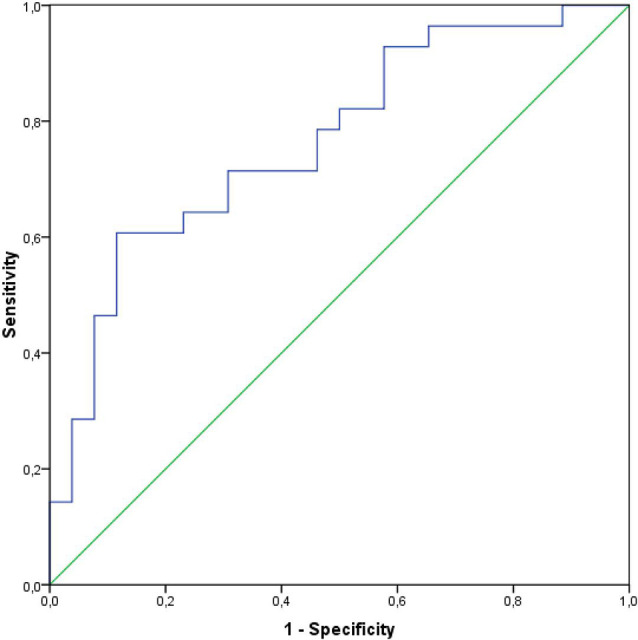
Receiver operating curves and the AUC for the CSF levels of S100B in a group of PD subjects.

### NSE

CSF NSE levels were significantly higher in PD patients as compared to healthy controls (mean ± SD-55.40 ± 7.17 vs. 45.88 ± 10.91 pg/ml; *p* = 0.00035; Figure 2).

No correlation was found between CSF NSE levels and, disease severity assessed either in H-Y scale or in UPDRS, or disease duration (*p* > 0.05; Table 2).

No significant correlations among PD subjects were found between CSF NSE levels and the patients’ age (*p* = 0.67), overall levodopa dose (*p* = 0.84) or levodopa equivalent (*p* = 0.95).

**Figure 4:**
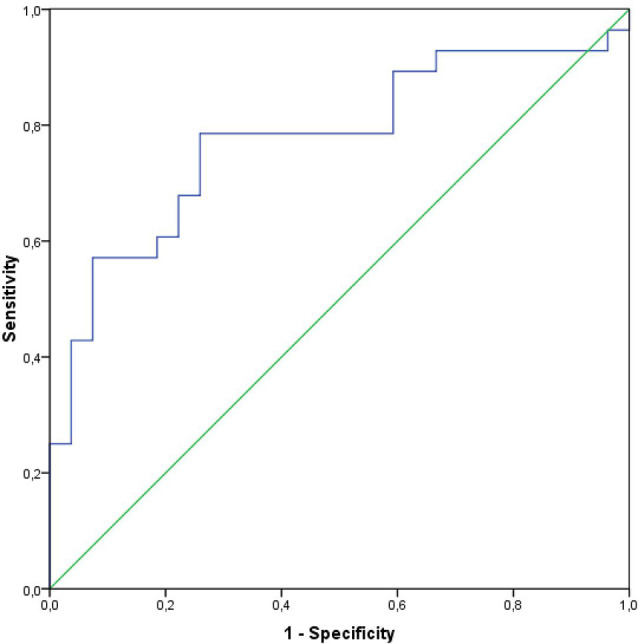
Receiver operating curves and the AUC for the CSF NSE in a group of PD subjects.

CSF NSE levels alone also provided a high discrimination value (AUC 0.775) between PD and healthy controls, with 78.6% sensitivity and 74.1% specificity (*p* < 0.001; Figure 4). A cut-off value to differentiate between PD and healthy subjects was 51.56 ng/ml (Youden index).

No correlations were found between NSE and S100B neither in PD subjects group nor in healthy control subjects (*p* = 0.785 and *p* = 0.746, respectively).

## Discussion

The aim of the present study was to clarify the role of CSF S100B as a marker of astroglial activation, and CSF NSE as a marker of neuronal damage in PD subjects. Presently the diagnosis of PD is made based on clinical evaluation, nevertheless there is a strong need for valid biomarkers, which could be characteristic of PD. In this study, we evaluated the levels of two CSF molecules, the one of glial and the second of neuronal origin, to assess their diagnostic potential related to the distinction between PD and healthy control subjects. The measurement of neuronal and glial derived proteins, e.g., NSE and 100 B in CSF has gained attention in another neurodegenerative disorder, which is AD, so far (Peskind et al., 2001; Chaves et al., 2010; Schmidt et al., 2014; Christl et al., 2019).

It is worth noting here that in the studies, which assessed only peripheral, serum levels of these two biomarkers among PD patients, no differences have been found between PD subjects and healthy controls (Schaf et al., 2005).

### S100B

In addition to depositions of abnormal proteins, PD is connected with impaired monoaminergic neurotransmission, involving mainly dopaminergic, serotoninergic and norepinephrine systems. Additionally, the disease is connected with immune system activation, especially microglial activation. S100B is one of these inflammatory biomarkers which gained interest in the context of microglial activation, as this protein is a primary product of astrocytes (Persson et al., 1987). In this study we wanted to check whether S100B may be involved in pathophysiology of PD.

The main findings of our study related to S100B, was its elevated levels in CSF of PD patients in relations to healthy control subjects, and the significant positive correlation of CSF S100B and disease severity and disease duration.

Comparable levels of serum S100B in a large cohort of PD patients in relation to controls were found by Schaf, but no CSF S100B levels were assessed in that study (Schaf et al., 2005). Interestingly Schaf et al. (2005) found a positive correlation between serum S100B levels and disease severity (in H-Y scale), which is in line with our results. The increase of CSF S100B levels with increased disease severity (H-Y scale) and with longer disease duration seems to be a consequence of glial degeneration, which progresses with time. Since astrocytes are the main source of S100B, results of our study indirectly confirm that astrocytes may play a significant role in PD pathophysiology. Increased CSF S100B levels in PD subjects seem to reflect both neuroinflammatory astroglial and microglial activation.

Our results are in line with data presented by Sathe et al. (2012) where increased CSF S100B levels were found in 84 PD subjects as well as in substantia nigra of six PD subjects, on post-mortem examination. Additionally, Sathe et al. (2012) confirmed that genetic ablation of S100B reduces microglial response, which also supports the hypothesis that S100B protein may be involved in the neuroinflammatory process present in PD.

In the current study, the area under the receiver operating characteristic curve as a measure for the discrimination between PD and controls for S100B was 0.77, showing a moderate discriminative effect. Our results are in line with results of another study, where the area under curve for CSF S100B to differentiate between PD and healthy subjects was also 0.76 (Sathe et al., 2012).

It should be mentioned here, that CSF S100B may only be treated as a non-specific marker of glial activation and/or degeneration in PD, taking into account its moderate discrimination ability, but also the fact, that increased CSF S100B were also found in different other neurodegenerative disorders. As mentioned above, increased CSF S100B levels have also been found in in Alzheimer’s disease (Petzold et al., 2003), frontotemporal dementia (Petzold et al., 2003) or brain injury (Kleindienst et al., 2010).

At the same time, the above results suggest that S100B levels may have a potential role as non-specific marker of PD progression. As S100B CSF levels increase with more advanced disease severity, it could be potentially of interest as a biomarker of early and preclinical PD. Nevertheless, further studies on a larger group of PD patients, involving subjects in very early disease stages, could answer the question whether CSF S100B may be sensitive enough to detect early, prodromal phases of PD. Of course, as a non-specific marker of glial degeneration, it cannot serve as a single marker of degenerative process in PD, but rather in connection with other biomarkers of different underlying pathological processes present in PD. Nevertheless, it seems reasonable to add this typical inflammatory marker to the spectrum of established markers of PD progression (Hall et al., 2015; Delgado-Alvarado et al., 2017; Mollenhauer et al., 2017, 2019), to cover a broader spectrum of pathological processes present in PD. Until now, among CSF biomarkers only alpha-synuclein has been proven to correlate positively with disease severity assessed in H&Y scale, UPDRS part III and Timed up and Go test (Hall et al., 2015). Nevertheless, CSF alpha-synuclein as a PD biomarker has several important limitations, which hamper its clinical use. Firstly, α-synuclein CSF levels can be strongly increased by blood contamination, and this results in considerable heterogeneity between individual PD patients and between different studies with PD subjects (Parnetti et al., 2019).

### NSE

Results of our study revealed that CSF NSE levels were significantly higher in PD patients than in healthy control subjects (*p* < 0.05; Figure 2). Additionally, we confirmed that CSF NSE does not correlate significantly with markers of disease severity like H&Y score or UPDRS part III.

NSE is not physiologically secreted, thus its increase in serum or CSF confirms neuronal damage (Constantinescu et al., 2009). The intraneuronal NSE is secreted into the extracellular space only after substantial neuronal damage, and its levels increase with the progression of neurodegenerative disorder (Chaves et al., 2010). CSF NSE has not been widely assessed in PD so far. In some studies, its CSF levels were assessed in atypical parkinsonisms (Abdo et al., 2004, 2007). Abdo et al. found increased NSE levels in CSF of MSA subjects, which is in line with more widespread neuronal damage present in MSA (Abdo et al., 2004, 2007; Katayama et al., 2020).

Contrary to our results, Katayama et al. (2020) found comparable CSF NSE levels in PD group and in healthy control subjects. Nevertheless, in Katayama’s study the investigated group was not homogenous and included a limited number of PD patients (21 PD subjects) as well as six DLB subjects (Katayama et al., 2020).

Contrary to CSF S100B, CSF NSE does not seem to be a useful marker of PD progression, as no correlation was found between CSF NSE and disease severity (neither in H-Y scale nor in UPDRS) or disease duration. The reason for such results may also be a short disease duration of PD patients involved in our study. At earlier stages of the disease the neurodegenerative process is mainly limited to the midbrain, and is not widespread to the whole brain. It is possible that involvement of PD in later stages of the disease would change the results of the correlation analysis.

Interestingly, we found no correlations between CSF NSE and CSF S100B either in PD subjects’ group or in healthy control subjects. This could potentially be explained by pathological specificity of neurodegenerative processes present in PD. It is known that the loss of dopaminergic neurons observed in brains of PD patients is typically accompanied by a glial reaction (Teismann et al., 2003) but it is also known that some dopaminergic neurons are more vulnerable to this pathological process within the CNS (Hirsch et al., 1998). It has been hypothesized that glial cells surrounding dopaminergic neurons influence this selective dopaminergic neuron’s vulnerability, as there is an inverse relationship between the degree of neuronal loss within dopaminergic cell of the substantia nigra and the density of surrounding astroglial cells, which suggests that dopaminergic neurons degenerate more actively in areas with lower number of astrocytes (Damier et al., 1993). This suggests that the neurodegenerative process within neuronal and glial cells is not parallel and may potentially explain lack of correlations between these two CSF markers.

Since NSE seems to be a non-specific marker of neuronal damage in different neurological disorders, it cannot be treated as a single CSF biomarker to differentiate PD patients from controls. Nevertheless, the results of our study do show that increased CSF NSE probably reflect neuronal damage present in PD. The same applies to increased CSF S100B level in PD, which should be treated as non specific marker of glial damage in PD. Since increased CSF NSE and S100B levels showed a relatively high discriminatory value for differentiating between PD and control subjects, so their levels may be helpful in differential diagnosis, especially when used with other CSF biomarkers for PD (Parnetti et al., 2013; Oosterveld et al., 2019), as, when used alone, they reflect only one pathological process present in PD.

The limitations of our study need to be mentioned. The first limitation is limited number of participants. This is mainly due to the fact that lumbar puncture requires hospitalization, is not widely accepted by all patients and their families, and its performance does not directly influence the diagnostic process. Secondly, the diagnosis of PD subjects involved in the study was based on clinical symptoms, which is however a typical diagnostic approach in neurodegenerative departments.

## Conclusions

Presently, there is no single CSF biomarker which allows to differentiate between PD and healthy control subjects. This is partially due to different underlying pathological processes present in PD, but also due to spatially restricted neurodegenerative process present in PD, limited mainly to the midbrain, especially in the early stage of the disease. Nevertheless there is sufficient evidence for some CSF biomarkers, including α-synuclein, NFL and classical AD biomarkers, to be helpful in differential diagnosis of PD. But still the highest performance accuracy can be achieved while assessing a combination of biomarkers. Since no single CSF brain derived protein can alone differentiate PD from healthy subjects, that is why different other particles have to be considered and evaluated to find the proper biomarkers for PD.

Our results require confirmation on larger group of PD patient, in different disease stages, nevertheless both CSF S100B and CSF NSE seem to be promising markers of axonal and glial degeneration, respectively, as parallel processes present in PD.

## Data Availability Statement

The raw data supporting the conclusions of this article will be made available by the authors, without undue reservation.

## Ethics Statement

The studies involving human participants were reviewed and approved by Ethics Committee of Medical University of Lublin, Poland. The patients/participants provided their written informed consent to participate in this study.

## Author Contributions

EP: conceptualization, data collection, formal analysis, investigation, and writing—original draft. EP and KR: methodology, software, and writing—review and editing.

## Conflict of Interest

The authors declare that the research was conducted in the absence of any commercial or financial relationships that could be construed as a potential conflict of interest.
